# Melatonin and Oral Cavity

**DOI:** 10.1155/2012/491872

**Published:** 2012-06-25

**Authors:** Murat İnanç Cengiz, Seda Cengiz, Hom-Lay Wang

**Affiliations:** ^1^Department of Periodontology, Faculty of Dentistry, Zonguldak Karaelmas University, 67600 Kozlu, Zonguldak, Turkey; ^2^Department of Prosthetic Dentistry, Faculty of Dentistry, Zonguldak Karaelmas University, 67600 Kozlu, Zonguldak, Turkey; ^3^Department of Periodontics and Oral Medicine, School of Dentistry, The University of Michigan, Ann Arbor, MI 48109-1078, USA

## Abstract

While initially the oral cavity was considered to be mainly a source of various bacteria, their toxins and antigens, recent studies showed that it may also be a location of oxidative stress and periodontal inflammation. Accordingly, this paper focuses on the involvement of melatonin in oxidative stress diseases of oral cavity as well as on potential therapeutic implications of melatonin in dental disorders. Melatonin has immunomodulatory and antioxidant activities, stimulates the proliferation of collagen and osseous tissue, and acts as a protector against cellular degeneration associated with aging and toxin exposure. Arising out of its antioxidant actions, melatonin protects against inflammatory processes and cellular damage caused by the toxic derivates of oxygen. As a result of these actions, melatonin may be useful as a coadjuvant in the treatment of certain conditions of the oral cavity. However, the most important effect of melatonin seems to result from its potent antioxidant, immunomodulatory, protective, and anticancer properties. Thus, melatonin could be used therapeutically for instance, locally, in the oral cavity damage of mechanical, bacterial, fungal, or viral origin, in postsurgical wounds caused by tooth extractions and other oral surgeries. Additionally, it can help bone formation in various autoimmunological disorders such as Sjorgen syndrome, in periodontal diseases, in toxic effects of dental materials, in dental implants, and in oral cancers.

## 1. Introduction

Melatonin (N-acetyl-5-methoxytryptamine) was isolated and characterized in 1958 [1] although one of its actions, that is, its ability to blanch the skin of amphibians, had already been shown in 1917 [2]. Melatonin is synthesized by the pineal gland and a variety of other organs. Pinealocytes, the major cells of pineal gland, are responsible for producing and secreting melatonin into the blood. The mechanisms of melatonin synthesis are well known and have been described in numerous publications [3–6]. Briefly, the pinealocytes take up free tryptophan from the blood and convert it to serotonin, which involves the enzymes tryptophan-5-hydroxylase and 5-hydroxytryptophan decarboxylase that successfully hydroxylate and decarboxylate tryptophan, respectively. At night, serotonin is converted to N-acetylserotonin by the action of N-acetyltransferase. Thereafter the enzyme hydroxyindole-O-methyl transferase acts on N-acetylserotonin causing its methylation and forming melatonin. In animals including man, melatonin reaches its maximal levels near the middle of the dark period with uniformly low levels during the day [7]. Because of the association of pineal melatonin synthesis with night time, melatonin is referred to as the chemical expression of darkness [8].

Among many actions, melatonin and its metabolites are highly effective free radical scavengers [9–11] and stimulators of antioxidative enzymes. Arising out of its antioxidative actions, melatonin protects cells during severe inflammatory processes and reduces oxidative damage [12]. Intense inflammatory processes contribute to the development of certain cancers, cellular damage caused by ionizing radiation, alterations in metabolism, and destruction of essential molecules and cells [3]. Melatonin also plays an immunomodulatory role [4], by regulating the secretion of interleukin-2 (IL-2) and interferon-alpha (INF-alpha) and the consequent activation of CD4+ lymphocytes [13]. Moreover, melatonin reportedly stimulates the proliferation and synthesis of type I collagen and promotes bone formation [14]. Once in the blood, melatonin also is discharged into the saliva. The proportion of plasma melatonin entering the mouth via the salivary glands appears to be relatively stable and ranges from 24% to 33%. It is widely agreed that 70% of plasma melatonin is bound to albumin. As only the free melatonin in plasma enters the saliva, salivary melatonin levels reflect the proportion of free-circulating melatonin [7]. Given the properties of melatonin and its presence in oral cavity because of this release in saliva [6], its effect on oral health warrants further investigation. 

Melatonin is nontoxic highly lipophilic indole, and this feature facilitates its penetration through cell membranes and its compartments. However, the most important effect of melatonin seems to result from its potent antioxidant, immunomodulatory, protective, and anticancer properties. Thus, melatonin could be used therapeutically, for instance, locally, in the oral cavity damage of mechanical, bacteria, fungal, or viral origin, in postsurgical wounds caused by tooth extractions and other oral surgeries, and in helping bone formation in various autoimmunological disorders, such as Sjogren syndrome, in periodontal diseases, in toxic effects of dental materials, in dental implants, and in oral cancers.

In this paper, we consider the potential roles of melatonin in the oral conditions including (1) tooth development and caries; (2) melatonin as an anti-inflammatory agent in oral cavity; (3) periodontal disease; (4) herpes viral infection; (5) candidiasis; (6) dental materials; (7) in oral cancers; (8) in dental implants. The paper provides a description and explanation of the clinical implications of melatonin in oral diseases. 

## 2. Melatonin in Tooth Development and Caries

Numerous studies have documented that melatonin is an important mediator in bone formation and stimulation [15]. Melatonin, with its antioxidant properties and its ability to detoxify free radicals, may interfere in this function of the osteoclast and thereby inhibit bone resorption [16]. The osteogenic effect of melatonin may be of clinical importance, as it could be used as a therapeutic agent in situations in which bone formation would be advantageous, such as in the treatment of fractures or of osteoporosis [16]. Melatonin may play a physiological role in tooth development/growth by regulating the cellular function of odontogenic cells in tooth germs [17].

In hamsters, a highly seasonal species, more caries lesions develop in the spring and summer, when the duration of nocturnal elevated melatonin is minimal, and, conversely, caries occur less commonly in the automan and winter when melatonin levels are maximal [18]. Moreover, it is known that tooth and osseous tissue material is strongly modified by cariogenic diets [18] and, given that melatonin is available in the foodstuffs, the quantity of melatonin in the consumed foodstuffs may influence caries incidence [19]. 

## 3. Melatonin as an Anti-Inflammatory Agent in Oral Cavity

The antioxidant properties of melatonin may be benefical for the treatment of the local inflammatory lesions and for accelerating the healing process, for example, after tooth extraction and other surgical procedures in the oral cavity [10]. Moreover, melatonin can be used to reduce inflammation produced as a side effect during therapy [3, 6]. Melatonin also has been shown to inhibit the inflammatory enzyme cyclooxygenase-2 (COX-2). Melatonin reportedly binds to the active sites of COX-1 and COX-2 indicating that it may act as a natural inhibitor of the function of these enzymes and thereby be an endogenous inhibitor of inflammation [20]. The anti-inflammatory and immunostimulatory actions of melatonin are well known [4].

 Melatonin has important physiological functions that have not been exploited in dentistry. The actions of melatonin, as described herein, may have clinical applications for improving the health of the oral cavity. This suggests that melatonin could be used therapeutically, for instance locally in the oral cavity diseases such as bacterial and viral lesions, postsurgical wounds, and oral surgeries, acting as a promoter of bone formation, as an important factor in autoimmunological disorders such as Sjorgen syndrome, in periodontal diseases, aphthous ulceration, Lichen planus, oral cancers, and even in toxic effects of dental materials [6].

## 4. Melatonin and Periodontal Disease 

Periodontal tissue is destroyed in the course of periodontitis by disproportionate immunologic responses to a triggering agent such as bacteria in biofilm [21]. Free radicals burst from the phagocytic cells, such as neutrophils and macrophages and migrate to the inflammation place and significantly damage the gingival tissue [22]. Lipid peroxidation is a major factor in the induction and progression of chronic periodontitis [23]. Increased reactive oxygen species (ROS) captured by melatonin and its metabolites in the inflamed area would be beneficial in reducing the degree of tissue damage. Moreover, melatonin influences fibroblast activity and bone regeneration by promoting osteoblast differentiation and bone formation [24]. Melatonin also stimulates the synthesis of type I collagen fibers [25]. It has been established that melatonin mediates these effects through receptor localized on preosteoblasts which leads to the production of bone sialoprotein, alkaline phosphatase, osteopontin, and osteocalcine in these cells, thus significantly shortening the time needed for their differentiation into mature osteoblasts from 21 to 12 days [9].

The receptor activation of nuclear factor-kappa B ligand (RANKL) is an important protein in osteoclastic differentiation and proliferation [26]. Another protein, osteoprotegerin (OPG), interferes with its biologic potential. RANKL and OPG play critical roles in the development of periodontal disease, with periodontal bone destruction resulting from the upregulation of RANKL with downregulation of OPG [27]. Melatonin alters these events by modulating the molecular triad of OPG/RANK/RANKL [28]. Also, treatment with melatonin stimulates the proliferation, differentiation, and activity of osteoblasts. Moreover, melatonin may act at the level of the osteoclasts lacuna, because of these antioxidant properties and its ability to neutralize reactive species where it inhibits bone resorption [29]. There is some evidence that topical application of melatonin may act as a biomimetic agent in the placement of endoasseous dental implants [14]. Because of the antioxidant and anti-inflammatory effects of melatonin, an increase in salivary melatonin levels may improve the organism's defensive response to the periodontal inflammatory process. A recent study [30] showed that melatonin strongly suppresses nitric oxide (NO) and interleukin-6 (IL-6) production induced by lipopolysaccharide (LPS) from *P. intermedia*, a major cause of inflammatory periodontal disease, in macrophages. Few studies showed both saliva [24, 31] and Gingival Crevicular Fluid (GCF) [31] melatonin levels, indicating that melatonin may have a protective role against periodontal disease. As the degree of periodontal disease increased, the salivary and GCF melatonin level decreased, indicating that melatonin acts to protect the body from external bacterial insults [24, 31]. The administration of melatonin, in local or systemic form, might be indicated in these patients, with the goal to protect their mouth against inflammatory and infectious processes of diverse nature.

## 5. Melatonin and Herpes Viral Infection 

The benefical effects of melatonin in herpes infections of the oral cavity have been compared with Acyclovir. In this case, melatonin proved benefical in reducing the severity of herpes at least as effectively as the prescription drug. This is consistent with the actions of melatonin on other viral infections where it has also been found to reduce the severity of those infections. The benefits of melatonin in these situations seem to stem from the immunomodulatory actions of melatonin in the stimulation of IL-1B, which has antiviral effects. The suppressive actions of melatonin on herpes may also relate to its stimulation of NK, CD4 cells, and so forth. At this point, the precise mechanism where by melatonin may reduce the severity of herpes infections remains unknown [32]. 

To promote the regression of the symptoms of herpes virus infection, a formulation containing 2.5 mg melatonin and 100 mg SB-73 (a mixture of magnesium, phosphate, fatty acids, and protein extracted from *Aspergillus oryzae*) with no reported side effects has been developed. This formulation is based on published information indicating that melatonin has known immunomodulatory properties. As it is considered a supplement, with natural ingredients, it may be fine utility in the treatment of herpes infections by individuals who cannot afford prescription drugs. Other studies have documented the antiviral actions of melatonin [33]. Currently known effects of SB-73 on immune system components include stimulation of production of T lymphocytes and cytokines, particularly interleukin-2 (IL-2) and interferon-gamma (INF-gamma) leading to increased activity of natural killer (NK) cells. SB-73 given either before or after viral infection increased the number of bone marrow granulocyte-macrophage progenitor cells (CFU-GMs) [32, 34]. As it is known that IFN-gamma has immunoregulatory, antiviral activities, it may be hypothesized that the action of SB-73 involves immunotherapeutic one.

## 6. Melatonin and Candidiasis

As an immunomodulator, melatonin reportedly exhibits protective effects in severe sepsis/shock induced by bacterial lipopolysaccharide in animal models. Melatonin reduced IL-6 levels and shortened time to improvement in animals with *Candida sepsis*. Levels of TNF-alpha and adhesion molecules in melatonin-treated septic rats were reduced compared with those in untreated septic rats [35]. Considering these findings, melatonin may have therapeutic benefits in Candida sepsis and classic antimycotic treatment because of this immune-regulatory effects. Thus, melatonin may also be useful as a topical and/or systemic treatment of oral candidiasis. 

Terron et al. [36] evaluated the effect of melatonin on the ingestion and destruction of *Candida albicans* (live particles) by the ring dove (*Streptopelia risoria*) at different durations of incubation with physiological as well as with a pharmacological concentration of melatonin. Also, some study results support the proposal that melatonin enhances phagocytic function and at the same time reduces oxidative stress originating during candidiasis [36, 37].

## 7. Dental Materials and Melatonin

Contemporary dental restorative materials mainly consist of methacrylate polymers with some additives. However, because of the incompleteness of polymerization process in situ as well as mechanical shearing and enzymatic degradation, methacrylate monomers are released from the restoration into the oral cavity and the pulp, from where they gain access to other tissues and organs. Such monomers have displayed toxic properties in many in vivo and vitro studies including cytotoxicity and genotoxicity and a considerable portion of these effects is underlined by the oxidative action of these compounds [38, 39].

Methacrylate compounds are widely used in polymeric forms in restorative and esthetic dentistry as well as in orthodontics [40]. However, the polymerization process, which occurs in situ, is always incomplete, resulting in the presence of methacrylate monomers in the oral cavity and the tooth itself, where they can migrate through dentinal microchannels to enter the pulp cavity; thereafter, they gain access to the vascular system and methacrylate monomers may be potentially present in all tissues and organs after being transported in the bloodstream.

The monomers have been reported to induce a broad spectrum of adverse biological outcomes, including cytotoxic and genotoxic effects [39]. On the other hand, melatonin shows a biocompatibility with tissues of the oral cavity and, because of its multiple antioxidative, anti-inflammatory, and oncostatic actions, it could be considered as a protective agent against harmful effects of dental materials [41–45].

## 8. Cytotoxicity and Genotoxicity of Dental Materials 

Traditional amalgam dental fillings, because of their high content of toxic mercury, have been replaced by dental composite materials, which are a mixture of organic polymers with inorganic fillers. Usually, these polymers are formed in situ from methacrylate-acid-(MAA-) based monomers: 2-hydroxymethyl methacrylate (HEMA), bisphenol A-diglycidyl dimethacrylate (bis-GMA), urethane methacrylate (UDMA), triethylene glycol dimethacrylate (TEGDMA), methyl methacrylate (MMA), and others. A considerable fraction of monomers does not undergo polymerization, and monomers, comonomers, and other substances may be released from polymerized materials because of a mechanical stress associated with chewing and the action of enzymes present in the saliva. Moreover, polymethacrylates may contain hydrolyzable ester groups at their surface and the action of esterases may release products of degradations of monomers into the oral cavity. MAA-based monomers are esters, so they can be targeted by esterases when in the organism and undergo dissolution [46].

Degradation of polymers may be induced also by bacteria, especially when the composite filling is affected by polymerization shrinkage, creating a gap between the filling and dentin [47]. Methacrylate monomers may diffuse through the dentinal tubules and reach miolimolar concentrations in the pulp, where they can damage pulp cells and migrate further into the bloodstream [48, 49]. As a consequence, HEMA leaching from dentin adhesives may reach concentrations in the pulp as high as 1.5–8 mM [43]. Likewise, TEGDMA concentrations reaching the pulp range up to 4 mm [20]. These concentrations are sufficiently high to induce a wide spectrum of harmful effects in pulp cells and other tissues, which can be reached by methacrylates from the bloodstream. 

Also, it has been shown recently that methacrylate monomers used in dentistry could interact with DNA of human lymphocytes, inducing single- and double-strand breaks and alterations to the DNA bases, including their oxidative modifications [50]. Therefore, methacrylate monomers used in restorative and esthetic dentistry may have the potential for inducing genotoxic effects. Phenotypic changes studies on the mechanisms of these effects may help to prevent or diminish their consequences. Oxidative mechanisms underlying some of these effects suggest the use of well-recognized antioxidants in a prevention strategy. Melatonin is one such antioxidant and its metabolites are also potent antioxidants [3, 5, 51].

## 9. Protective Action of Melatonin against DNA-Damaging Agents 

Melatonin has a long history of studies documenting its ability to prevent DNA damage induced by toxic chemicals and ionizing radiation [5, 52]. Also, results of many studies performed in vitro suggest protective effects of melatonin in normal cells against several agents present in the environment, including lead [53, 54], arsenic and fluoride, both singly and in combination [55, 56]. In the latter study, melatonin also inhibited sister chromatid exchanges and stimulated cell proliferation.

Reactive oxygen species are implicated in induction of programmed cell death, that is, apoptosis [57]. On the other hand, melatonin displays strong antioxidative properties and it is a potent antiapoptotic agent. The mechanisms whereby melatonin regulates the apoptotic program are being intensively investigated [57–59]. Therefore, melatonin's action as a scavenger of reactive oxygen species and its involvement in the repair of the damage mediated by these species may be considered.

## 10. Biocompatibility of Melatonin in the Oral Cavity 

Melatonin is present in the oral cavity as a consequence of its release into the saliva. A significant correlation between concentrations of melatonin in saliva and serum was reported with the general conclusion that melatonin concentration is a reliable index of serum melatonin level [24, 29].

Melatonin may play an important role in many physiological processes in the oral cavity and decrease the conseqences of several pathologies [29].

The biocompatibility of melatonin with the tissues of the oral cavity, the need of defense against cytotoxic, genotoxic action of methacrylate-based dental materials as well as the current results suggesting compliance of melatonin with dental materials and its protection against harmful effects induced by these materials clearly justify further research on the application of melatonin in dentistry. 

Melatonin decreases cytotoxic and genotoxic effects of methacrylate monomers used in dentistry, and it does not influence the bond strength of dental composites. This opens a new possible application of melatonin to improve properties of biomaterials used in dentistry. 

## 11. Melatonin and Oral Cancer

Melatonin exerts oncostatic activity through several biologic mechanisms including antiproliferative actions, stimulation of anticancer immunity, modulation of oncogene expression, and anti-inflammatory, antioxidant, and antiangiogenic effects [60]. Melatonin inhibits human cancer cell growth in culture and preliminary clinical studies seem to confirm its anticancer property in vivo as well. In addition, melatonin may have other biologic effects, which could be useful in palliative therapy for cancer, namely, anticachectic, antiasthenic, and thrombopoietic properties [61]. Melatonin appears to a promising anticancer agent [62]. First, melatonin scavenges ROS, which are known second messengers in the signaling pathways leading to the cell division [60]. Additionally, melatonin amplifies the antitumor activity of interleukin-2 [62]. Melatonin is a proven powerful cytostatic drug in vitro as well in vivo [63–65]. Melatonin may be successfully administered in medical oncology in the suppurative care of untreatable advanced cancer patients and for the prevention of the side effects of chemotherapy.

More recent studies have shown that melatonin functions are much more complex than those exclusively related to circadian and circannual cycles. They point to an association between aspects of intracellular functions and melatonin that involve mechanisms independent of its action on receptors. Melatonin is just one of the many factors that can control cell proliferation. In this group, melatonin is the only known chronobiotic and hormonal regulatory of neoplastic cell growth. With physiological circulating concentrations, melatonin is cytostatic and inhibits cancer cell proliferation [66].

Melatonin is the circadian signal that mediates seasonal reproductive-cycle variations in photoperiodic mammals and influences numerous aspects of circadian rhythms by binding to membrane receptors [67]. The production and release of nearly all hormones exhibits diurnal timing patterned on an approximately 24 h cycle. Lifestyle factors (e.g., working a night shift or sleep disruption) or exposure to specific agents (e.g., light at night (LAN)) disrupts circadian rhythm or alters endocrine function and, possibly, the regulation of reproductive hormones relevant to the etiology of hormone-related diseases like breast or prostate cancer [68]. Hence, individuals affected by lack of continuous light, for example, the blind, are un affected by the melatonin-production inhibition that affects night workers. Breast tumors are less prevalent in blind women [69]. In 2007, IARC (WHO) corroborated this by classifying “shift-work that involves circadian disruption” as potentially carcinogenic [70]. 

In relation to oral cancer, it has been speculated that exogenous restoration of melatonin receptor 1 A (MTNR1A) expression inhibited the growth of oral squamous cell carcinoma cells lacking its expression [71]. Together with the known tumor-suppressive functions of melatonin and the presence of MTNR1A in various tumors, the MTNR1A is likely target for epigenetic silencing at loci 4q35 and may play a pivotal role during oral carcinogenesis [72]. In precancerous oral diseases such as leukoplakia [21] and lichen planus, reactive species also are involved in their pathogenesis [71]. In light of these studies, the importance of melatonin in carcinogenesis in the oral cavity is clear. Melatonin released into the oral cavity via the saliva may have yet to be identified for oral health.

## 12. Melatonin and Dental Implants 

A variety of substances have been used to enhance peri-implant bone response: growth factors [73], morphogenetic proteins [74], and, more recently hormones, such as growth hormone, and melatonin [75]. A number in vitro studies have reported that melatonin is an important mediator in bone formation of stimulation, promoting osteoblast differentiation [76, 77]. Links between melatonin and bone metabolism have been documented in many studies [9, 76, 78]. In these investigations, melatonin acted on the bone as a local growth factor, with paracrine effects on nearby cells [79–81]. Also, it has been shown that melatonin influences precursors of bone cells in bone marrow of rats [80]. In addition, the indolamine has been found to be a significant modulator of calcium metabolism and reportedly prevents osteoporosis and hypocalcemia in certain cases, probably due to its interaction with other bone regulatory factors, such as parathormone, calcitonin, or prostaglandins [81–83]. These findings likely have biological significance and support some findings. Four weeks after implant insertion, melatonin with collagenized porcine bone significantly increased the osteointegration and reduced crestal bone resorption formation compared with porcine bone alone [84]. In general, bone substitutes have allo or xenogeneic origins or are synthetically made from calcium-based materials. Xenogeneic biomaterials display a similar morphology as human bone and have the potential of being resorbed. Deproteinized bovine bone is one of the most well-documented bone substitutes. This material has been shown to have osteoconductive properties and histological examination has demonstrated osseointegration of titanium implants in areas previously treated [84–86]. Recently, bone substitutes of porcine origin have been used for maxillary sinus floor augmentation prior to implant placement [84, 87]. 

It is also shown that bone-to-implant contact (BIC) and total peri-implant bone area at 4 and 12 wk are higher in melatonin-treated implant than in controls consistent with a previous study [84, 88]. The larger amount of bone tissue in direct contact with the implants that received topical melatonin perhaps reflects greater synthesis of bone matrix in the peri-implant area: this may be due to either an increase in the number or in the activity of osteoblast cells, or to the inhibition of osteoclast activity, or to both actions. One important function of melatonin seems to be the formation of bone cells. In this regard, several studies have shown that melatonin stimulates proliferation and differentiation of human osteoblasts in vitro, as well as the synthesis of type I collagen and other proteins of the bone matrix [76, 78, 80]. Also, newly formed bone and abundant bone trabeculae in direct contact with implant surfaces were observed after the administration of melatonin and recombinant human fibroblast growth factor-2 (FGF-2) by intraperitoneal injection in female rats [89]. Melatonin would appear to contribute to the formation of new bone around implants as it stimulates the differentiation of new preosteoblasts, which are transported from bone marrow to the alveolar bed via the vascular system, another action of melatonin at the preosteoblast level, which enhances new bone tissue formation by stimulation of gene expression of certain proteins in the bone matrix [88]. 

Macrophages and leukocytes from peri-implant blood vessels promate an increase in free radicals, which stimulate bone resorption by the osteoclast [10, 29, 90]. Melatonin is antioxidant and anti-inflammatory properties may attenuate this reaction and constrain the production of reactive species and therefore bone resorption, after implant surgery [91]. The inhibition of bone resorption may be reinforced by another reaction induced by melatonin on the osteoclastogenesis process.

These actions of melatonin on bone tissue are of interest as it may be possible to apply melatonin during endosseous dental implant surgery as a biomimetic agent [82]. As a result, the process of healing may be more precise, initial conditions of receptor tissues may be enhanced, the period of osteointegration and settling of the implant may be reduced, and therefore the quality of life of the patient may be improved.

## 13. Conclusions

While the inflammatory and oxidative mechanisms of the oral diseases are being unraveled, the use of novel anti-oxidant and anti-inflammatory substances is warranted. Melatonin may have clinical applications in reducing oral diseases, limiting tissue damage that is a result of free radicals, stimulating the immune response, reducing the progressive loss of alveolar bone, promoting the regression of symptoms of herpes viral infection, impeding local inflammatory lesions, and possible treatment of xerostomia and oral cancer also with the biocompatibility of melatonin with the tissues of the oral cavity and the need of defense against cytotoxic and genotoxic action of methacrylate-based dental materials. 

Melatonin has the following positive aspects: it is endogenously produced, it is nontoxic, it diffuses rapidly into all cells and body fluids, it penetrates all subcellular compartments, it is generally devoid of pro-oxidant actions, and it stimulates a number of antioxidant enzymes. Melatonin released into the oral cavity via the saliva may have yet-to-be identified benefits for oral health. Melatonin from the blood into the saliva may play an important role in suppressing oral diseases. It may have beneficial effects in periodontal disease, herpes, and oral cancer, amongst others [92]. Individuals, as the result of pathologies that are characterized by a malfunction of the salivary glands, may have an elevated capacity to develop diseases of oral cavity. The administration of melatonin, in local or systemic form, might be indicated in these patients, with the goal of protecting their mouth against inflammatory and infectious processes of a diverse nature. The functional aspects of melatonin in the oral cavity need additional investigating and may prove to be a fertile area for research. If melatonin has an effect in improving any aspect of oral health, the regular use of currently available sublingual tablets may be found to be useful as a means of treatment.
